# SpermTree, a species-level database of sperm morphology spanning the animal tree of life

**DOI:** 10.1038/s41597-022-01131-w

**Published:** 2022-01-31

**Authors:** John L. Fitzpatrick, Ariel F. Kahrl, Rhonda R. Snook

**Affiliations:** Department of Zoology, Stockholm University, Svante Arrhenius väg 18B, SE-10691 Stockholm, Sweden

**Keywords:** Sexual selection, Phylogenetics, Reproductive biology

## Abstract

Sperm are the most morphologically variable cell type known, despite performing the same functional role of fertilizing eggs across all sexually reproducing species. Sperm morphology commonly varies among individuals, populations, closely related species, and across animal phyla. Sperm morphology has long been used as a tool for placing species in a phylogenetic context and a range of selective forces are hypothesized to influence sperm evolution and diversification. However, we currently lack robust examinations of macroevolutionary (i.e. across phyla) patterns of sperm evolution, due largely to the challenges of comparing sperm morphological data across the animal tree of life. Here we describe the SpermTree database, which currently represents 5,675 morphological descriptions of sperm morphology from 4,705 unique species from 27 animal phyla. This dataset includes measurements of sperm head, midpiece, flagellum and total length, the latter of which spans four orders of magnitude. All entries in the dataset are matched to currently accepted scientific names in taxonomic databases, facilitating the use of these data in analyses examining sperm evolution in animals.

## Abstract (summary)

**Table:** 

Measurement(s)	sperm morphology trait • Spermatozoon
Technology Type(s)	Microscope Device
Sample Characteristic - Organism	Metazoa

Machine-accessible metadata file describing the reported data: 10.6084/m9.figshare.17151347

## Background & Summary

Sperm are one-half of the story of life for sexually reproducing animals, for which the fusion of sperm and eggs is necessary for the production of offspring. Yet, despite their shared function of fertilizing eggs, sperm are the most diverse cell type known, exhibiting large variation in size across animals, including examples of sperm ‘gigantism^1–4^’. A range of hypotheses have been developed to explain the tremendous diversity in sperm morphology. Generally, sperm morphology is hypothesized to be shaped by the environment in which sperm operate and where fertilization takes place (i.e. the fertilization environment^4,5^), a species’ evolutionary history (i.e. phylogenetic effects e.g.^6^), and the postcopulatory sexually selective forces of sperm competition and cryptic female choice^2,3,7–10^. Numerous studies have tested these hypotheses among closely related species^2,8–10^. However, we know far less about the factors shaping sperm morphology among different phyla^4^.

The thousands of descriptions of sperm morphology currently in the literature have yet to be systematically compiled in a single location. This stands in contrast to recently published databases on egg morphology in birds, amphibians, and insects^11–14^. The lack of a centralized repository of sperm morphology makes examining broad-scale evolutionary questions, drawing comparisons among phyla, and statistically identifying common (or distinct) evolutionary responses in sperm morphology challenging. To address these challenges, we generated a quantitative and descriptive dataset of sperm morphology parameters compiled from the literature, called the SpermTree database^15^.

**Fig. 1:**
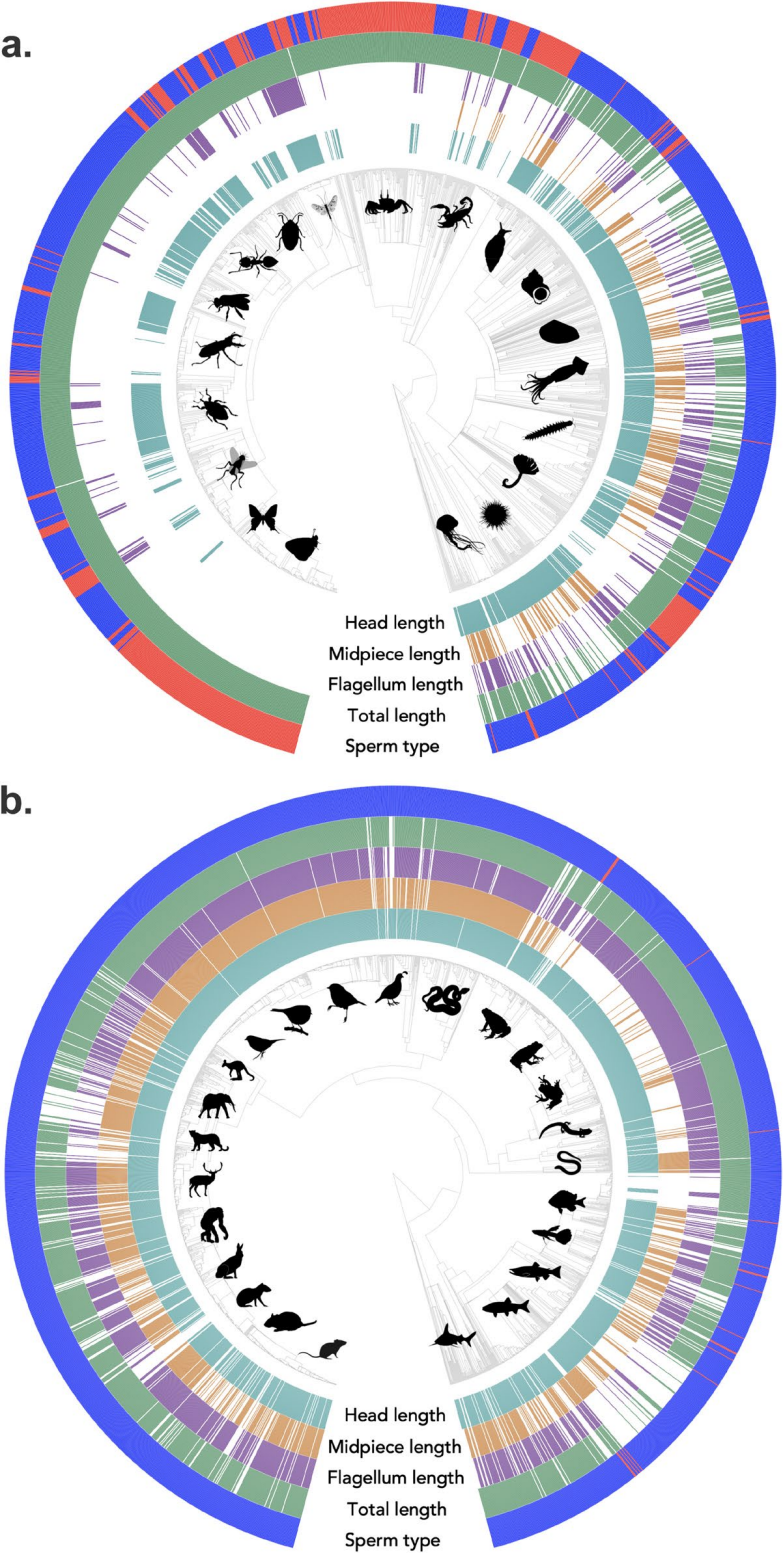
Distribution and availability of sperm morphology data for (**a**) invertebrate taxa and (**b**) the vertebrate taxa from the SpermTree database. To allow better taxonomic resolution in the figure, we show invertebrate and vertebrate taxa separately. In the central phylogenies, each tip represents a single species. The inner four rings around the phylogenies depict the presence (coloured bar) or absence (white bar) of data for sperm head length (cyan), midpiece length (orange), flagellum length (purple) and total sperm length (green) trait in the SpermTree dataset. The outer ring depicts the classification of sperm type for each species, with species with standard sperm morphologies coloured blue and species with non-standard sperm morphologies coloured red. Silhouette images provide examples of the taxonomic groups represented in our dataset and their relative phylogenetic placement. Silhouette images were obtained from PhyloPic (http://phylopic.org) under a public domain license (CC0 1.0 license).

**Fig. 2:**
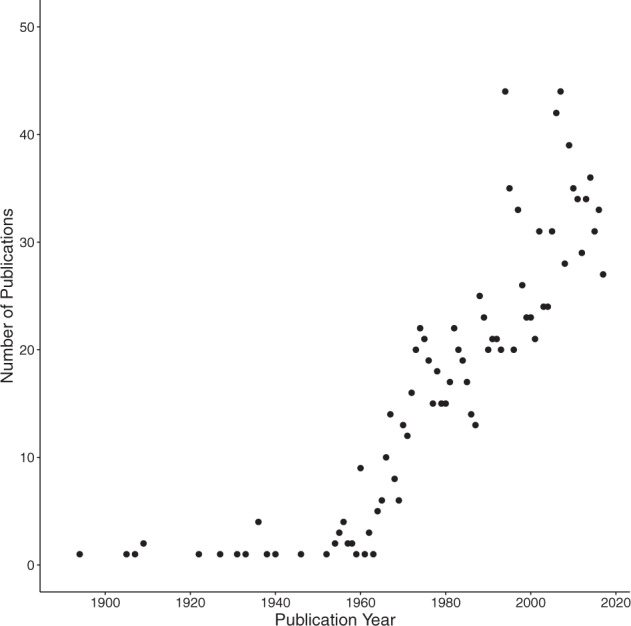
Publications on sperm morphology plotted by year. The number of publications in the SpermTree database are presented for each year. The primary sampling period was conducted up until 2018 as part of Kahrl *et al*.^4^, therefore this graph depicts years with complete sample records. Additional sources have been added since that publication, resulting in a database that spans 127 years.

**Fig. 3:**
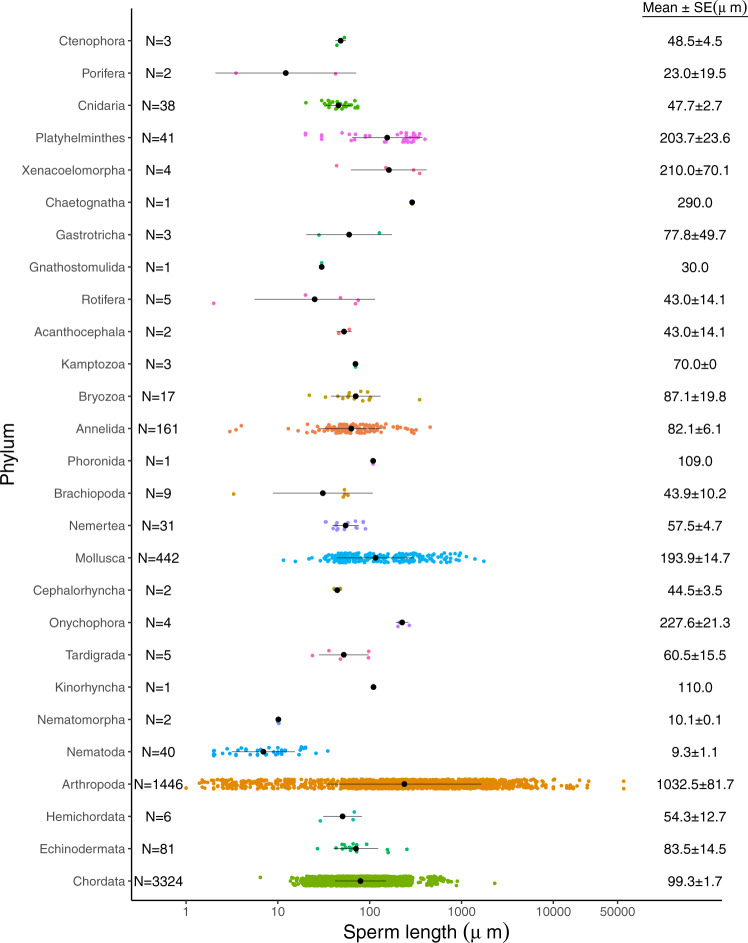
Within- and among-phyla variation in sperm length in species present in the SpermTree database. Sperm total length measurements (μm presented on a log_10_ scale) for species from each of the 27 phyla in the SpermTree database are presented. The figure depicts total sperm length as this sperm measure was available for the greatest number of species in the dataset (Table 1). Each coloured point indicates the total sperm length value from an individual species. Sample sizes (N) are presented for each phyla. The phyla mean and standard deviation are presented with a black circle and line, respectively. Mean (±SE) values are also provided for each phyla.

To build this dataset, we searched the literature, including journal articles, books, and monographs. Thus, the dataset includes records that are relatively straight forward to obtain (e.g. recently published papers) as well as those from more obscure, harder to obtain references (e.g. from books and dissertations that are not part of most library collections). This search process generated a dataset derived from 1,323 publications that currently includes species-level descriptions of 5,675 entries from 4,705 unique species in 27 animal phyla (Fig. 1). Data were obtained from published work over the past 127 years, with the number of publications increasing dramatically beginning in the early 1960’s (Fig. 2). There is both within- and among-phyla variation in sperm length values among the species of the 27 phyla included in the SpermTree database (Fig. 3).

The sperm morphology traits included in the SpermTree database are listed in Table 1. Entries include species with ‘standard’ sperm morphology (n = 4,914), where sperm are made up of a head, midpiece (if present) and a single flagellum. In addition, entries with ‘non-standard’ sperm morphology are also included in our dataset, including species with bi- or multi-flagellate sperm (n = 36), aflagellate sperm (n = 185), heteromorphic sperm (n = 375), or sperm with morphologies that defied standard classifications (e.g. sperm were encysted, pyriform, elongate but without a true flagellum, n = 165). The dataset also includes the scientific name used in the original publication, as well as currently recognized scientific names that have been matched to each species using the Catalogue of Life (www.catalogueoflife.org). This dataset is publicly available for download and represents an expanded version of a recent evolutionary analysis of how sperm size is influenced by fertilization mode described in Kahrl *et al*.^4^.

**Table 1:** Entries for sperm morphology traits obtained from published sources and included in the SpermTree database.

Sperm Trait	Number of entries
Sperm head length	4,095
Sperm midpiece length	2,067
Sperm principle piece length	1,403
Sperm flagellum length*	2,856
Sperm total length*	4,827
Sperm morphology type (standard/non-standard)	5,675

The number of entries in the dataset for each sperm trait are listed. *When not directly provided in the source publications, length was calculated by summing the relevant constituent sperm traits to obtain a length value.

Whereas the database includes entries from 27 animal phyla, the coverage across the animal tree of life is unbalanced (Figs. 1, 3). Vertebrates (from the phylum Chordata) represent more than half of the entries in the dataset (n = 3,295, Fig. 1a). The remaining entries (n = 2,380) are made up of invertebrates, with 61% of invertebrates represented by entries in the phylum Arthropoda (Figs. 1b, 3).

The SpermTree database facilitates a range of macroevolutionary analyses examining the evolution of sperm morphology in animals. For example, information in the database, coupled with data on social/sexual/ecological traits, can be used to examine: *i)* if rates of sperm evolutionary diversification are variable among sperm components, phyla, or environments *ii)* whether post-copulatory sexual selection, including sperm competition and cryptic female choice, influence the evolution of sperm morphology and whether such effects differ among phyla, *iii)* what factors influence the evolution of non-standard sperm morphologies, and *iv)* phylogenetic patterns of sperm morphology.

## Methods

Data on sperm head, midpiece, principle piece, flagellum and total length, along with classifications of sperm morphology type (i.e. standard vs. non-standard morphology) were compiled from the primary scientific literature, books, and online databases. For sperm morphology traits, online search engines (e.g. Google Scholar, Web of Science) we searched for combinations of Phylum, Class, Order, Family, and common names with the search terms ‘sperm morphology’, ‘sperm ultrastructure’, ‘sperm length’, ‘sperm design’, ‘sperm dimensions’, ‘spermatogenesis’, and ‘sperm morphometry’. We also repeated these search terms substituting ‘gamete’ or ‘spermatozoa’ in place of ‘sperm’. Where available, data were also obtained from relevant references listed in the literature that matched our search criteria.

## Data Records

### Data record 1: SpermTree

The raw data for the SpermTree database is available in xlsx file format at the Open Science Framework^15^ (uploaded June 29, 2021), including a separate sheet explaining the column headings. This file contains columns listing taxonomic classifications (e.g. Phylum, Class, Order, Family, Genus and Species) along with more commonly used classifiers (e.g. vertebrate vs. invertebrate) to facilitate partitioning of the dataset based on the needs of individual research projects. Additional notes about sperm morphology are provided where relevant. The SpermTree database can be found at OSF^15^.

### Data record 2: SpermTree, a living database

The current SpermTree database is a starting point for compiling sperm, and other relevant reproductive, data. The SpermTree database will be regularly updated and is publicly available on https://spermtree.org. This website allows additional data to be sent to authorized administrators for incorporating into the database, and incorrect records to be amended or deleted. Individuals wishing to contribute additional data or correct existing entries should go to https://spermtree.org for information on how to contact the site administrators.

This article is licensed under a Creative Commons Attribution 4.0 International License, which permits use, sharing, adaptation, distribution and reproduction in any medium or format, as long as you give appropriate credit to the original authors and the source, provide a link to the Creative Commons license, and indicate if changes were made. The images or other third party material in this article are included in the article’s Creative Commons license, unless indicated otherwise in a credit line to the material. If material is not included in the article’s Creative Commons license and your intended use is not permitted by statutory regulation or exceeds the permitted use, you will need to obtain permission directly from the copyright holder. To view a copy of this license, visit http://creativecommons.org/licenses/by/4.0/.

The Creative Commons Public Domain Dedication waiver http://creativecommons.org/publicdomain/zero/1.0/ applies to the metadata files associated with this article.

## Technical Validation

All entries were manually curated to ensure record accuracy. After entry into the dataset by one person, all entries were checked by a second person. Finally, a sub-sample of the entries were spot checked by a different person from the person who originally entered or checked the data. Data were plotted as histograms and using correlations (e.g. sperm head length vs. sperm midpiece length) to identify potential inconsistencies. Any record that appeared to be inconsistent was rechecked against the original publication source to confirm that they were correctly entered. The online SpermTree database will be expanded as new information becomes available and will be corrected as required.

## Usage Notes

The data are available for download as a xlsx file from Open Science Framework (June 30, 2021)^15^ and from https://spermtree.org. These data may be viewed on their own using Microsoft Excel (or other comparable programs) and can be imported into R^16^ (or other comparable programs) for use in data analyses. Importantly, the SpermTree database is extendable. Additional extensions to this database are envisioned, including enriching the database with more reproductive traits, and adding both population-level and individual-level sperm morphology trait variation. This variation can also include information about where the populations occur geographically and experimental conditions under which individuals in a population may vary. Such information would facilitate future analyses associating environmental variation and sperm-related reproductive trait variation and linking sperm morphological variation with potential agents of selection. Such additions would extend the potential of the SpermTree database for research aimed at understanding the evolution of sperm diversity. There are no restrictions on the re-use of this data. We request that researchers using the SpermTree database provide details of any resulting publications so we can post this information on the SpermTree website.

## Data Availability

The R code used to visually summarize the data in the current study is available at OSF^15^.
